# Synergistic Effects of *Bacillus amyloliquefaciens* (GB03) and Water Retaining Agent on Drought Tolerance of Perennial Ryegrass

**DOI:** 10.3390/ijms18122651

**Published:** 2017-12-11

**Authors:** An-Yu Su, Shu-Qi Niu, Yuan-Zheng Liu, Ao-Lei He, Qi Zhao, Paul W. Paré, Meng-Fei Li, Qing-Qing Han, Sardar Ali Khan, Jin-Lin Zhang

**Affiliations:** 1 State Key Laboratory of Grassland Agro-ecosystems, College of Pastoral Agriculture Science and Technology, Lanzhou University, Lanzhou 730020, China; suay13@lzu.edu.cn (A.-Y.S.); niushq14@lzu.edu.cn (S.-Q.N.); liuyzh13@lzu.edu.cn (Y.-Z.L.); heal15@lzu.edu.cn (A.-L.H.); qzhao@lzu.edu.cn (Q.Z.); hanqq16@lzu.edu.cn (Q.-Q.H.); ali.khan13@lzu.edu.cn (S.A.K.); 2 Department of Chemistry and Biochemistry, Texas Tech University, Lubbock, TX 79409, USA; paul.pare@ttu.edu; 3 College of Life Science and Technology, Gansu Agricultural University, Lanzhou 730070, China; lmf@gsau.edu.cn

**Keywords:** *Bacillus amyloliquefaciens*, perennial ryegrass, water retaining agent, synergistic effects, drought tolerance

## Abstract

Water retaining agent (WRA) is widely used for soil erosion control and agricultural water saving. Here, we evaluated the effects of the combination of beneficial soil bacterium *Bacillus amyloliquefaciens* strain GB03 and WRA (the compound is super absorbent hydrogels) on drought tolerance of perennial ryegrass (*Lolium perenne* L.). Seedlings were subjected to natural drought for maximum 20 days by stopping watering and then rewatered for seven days. Plant survival rate, biomass, photosynthesis, water status and leaf cell membrane integrity were measured. The results showed that under severe drought stress (20-day natural drought), compared to control, GB03, WRA and GB03+WRA all significantly improved shoot fresh weight, dry weight, relative water content (RWC) and chlorophyll content and decreased leaf relative electric conductivity (REC) and leaf malondialdehyde (MDA) content; GB03+WRA significantly enhanced chlorophyll content compared to control and other two treatments. Seven days after rewatering, GB03, WRA and GB03+WRA all significantly enhanced plant survival rate, biomass, RWC and maintained chlorophyll content compared to control; GB03+WRA significantly enhanced plant survival rate, biomass and chlorophyll content compared to control and other two treatments. The results established that GB03 together with water retaining agent promotes ryegrass growth under drought conditions by improving survival rate and maintaining chlorophyll content.

## 1. Introduction

Drought as one of the major abiotic stresses has been weighing heavily against the agricultural productivity worldwide [1,2], since most of crops and forage plants grown to feed the global population are highly sensitive to drought [3]. Drought also induces severe desertification, with a progressive reduction of the vegetation cover coupled with rapid soil erosion in arid and semi-arid climatic regions [4,5]. Drought affects water potential and turgor in plants, resulting in the changes of physiological and morphological traits. Fresh weight and relative water content are two parameters commonly adopted to measure the impact of drought stress on plant growth [6]. Drought decreases plant chlorophyll content, which is directly related to photosynthesis rate [7]. Drought is also known to increase the reactive oxygen species (ROS) in plant cells, which are well recognized for lipid peroxidation and cell membrane deterioration, resulting in secondary oxidative stress [8,9]. Among various impacts of drought stress on plant growth, nutrient and water availability are mainly discussed [10,11].

Plant growth promoting rhizobacteria (PGPRs) are microorganisms associated with plant roots and can confer beneficial effects on the host plants [12]. Early research reported that the PGPR *Paenibacillus polymyxa* enhanced drought tolerance of *Arabidopsis thaliana* [13]. *Bacillus amyloliquefaciens* had been applied to several commercial crops and shown the remarkable effects on the increase in plant growth, disease resistance as well as salt and drought tolerance [14,15,16]. *B. amyloliquefaciens* strain GB03 enhanced growth and abiotic stress tolerance in *Arabidopsis* by emitting a complex blend of volatile organic compounds (VOCs) [17,18,19,20,21]. These VOCs activated differential expression of approximately 600 transcripts including genes related to cell wall modifications, primary and secondary metabolism, hormone regulation and stress response [19]. Recent studies reported that GB03 also promoted growth and salt tolerance in wheat (*Triticum aestivum*) [22], white clover (*Trifolium repens* L.) [23] and a halophytic grass *Puccinellia tenuiflora* [24].

Super absorbent hydrogels used as water retaining agents (WRA) in agriculture were formed from highly hydrophilic cross-linked polymers, which possess high water absorption capacity [25]. It was found that the hydrogels can mitigate soil erosion by reducing sediment and nutrient losses [26,27,28]. The hydrogels can also absorb water and nutrients and subsequently release them gradually [29,30,31]. In addition, Sojka et al. found that hydrogel promoted soil colonization of microorganism, including bacteria and mycorrhiza [32]. It increases the plant available water in the soil, which prolongs plant survival time under drought stress [33,34,35,36]. Hayat et al. proved that the administration of WRA induced substantial changes in soil physical properties by increasing saturation percentage while decreasing particle density and bulk density, so as to promote crop productivity [37]. 

Perennial ryegrass (*Lolium perenne* L.) is an important grass species for pasture, forage and turf in the world [38]. This cool-season grass species is native to Northern Europe, Asia and Africa and widely distributed in many temperate regions all over the world [39]. It has good turf quality with quick establishment [40]. Like many other turf grasses, however, it is also a drought sensitive species [41].

Although either *B. amyloliquefaciens* GB03 or WRA can enhance the plant performance under drought conditions, the combination effects of both of them have not been reported to date. The objective of this research was to investigate the synergistic effects of the beneficial soil bacterium strain *Bacillus amyloliquefaciens* GB03 and water retaining agent on drought tolerance of perennial ryegrass. Plants were subjected to drought treatments by stopping watering for 20 days. Plant survival rate and parameters related to plant biomass, photosynthesis, water status and leaf cell membrane integrity were assessed.

## 2. Results

### 2.1. B. amyloliquefaciens GB03 and WRA Promoted Ryegrass Growth under Drought Condition

**Figure 1:**
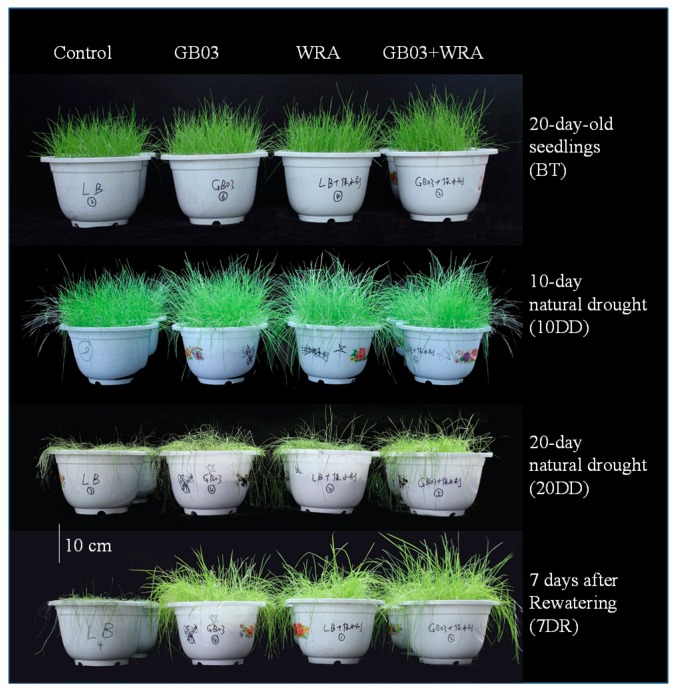
Ryegrass growth status of different treatments in four vegetation phases. From left to right: control, GB03, WRA and GB03+WRA treatments; from top to bottom: 20-day-old seedlings (before treatment, BT), 10-day natural drought (10DD), 20-day natural drought (20DD) and 7 days after rewatering (7DR).

**Figure 2:**
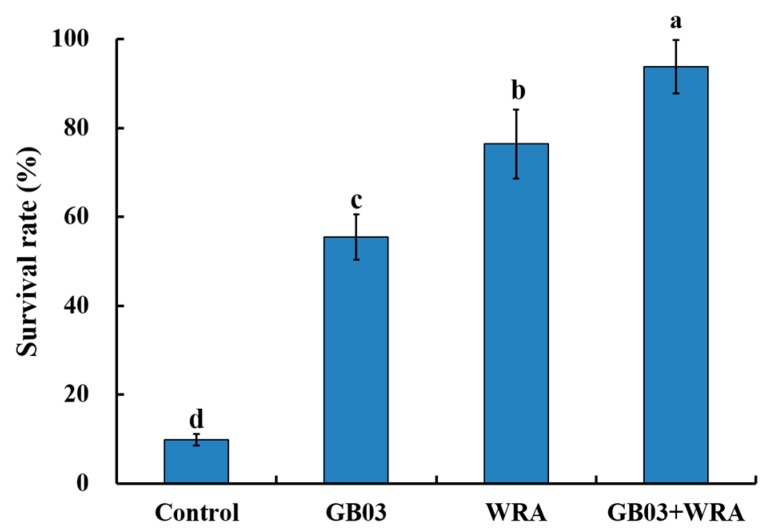
The survival rate of ryegrass seedlings with 20 days natural drought and then seven days after rewatering. Values are means and bars indicate standard errors (SEs) (*n* = 12). Columns with different letters indicate significant differences among treatments at *p* < 0.05 (ANOVA and Duncan’s post-hoc multiple comparison test).

Table 1 showed that the soil water contents of WRA and GB03+WRA treatments were higher than those of control and GB03 treatment. GB03 and WRA significantly increased leaf growth and plant density of ryegrass compared to control during all phases (*p* < 0.05) (Figure 1). No significant difference was observed among all treatments for 20-day-old (before drought treatment, BT) and 10-day natural drought seedlings. However, after 20 days natural drought and then seven days after rewatering, seedlings treated with GB03+WRA grew well and exhibited the highest survival rate (approximately 94%), compared with those treated with WRA (76%) that was significantly higher than that of seedlings treated with GB03 (55%) or control (10%) (Figure 2).

**Figure 3:**
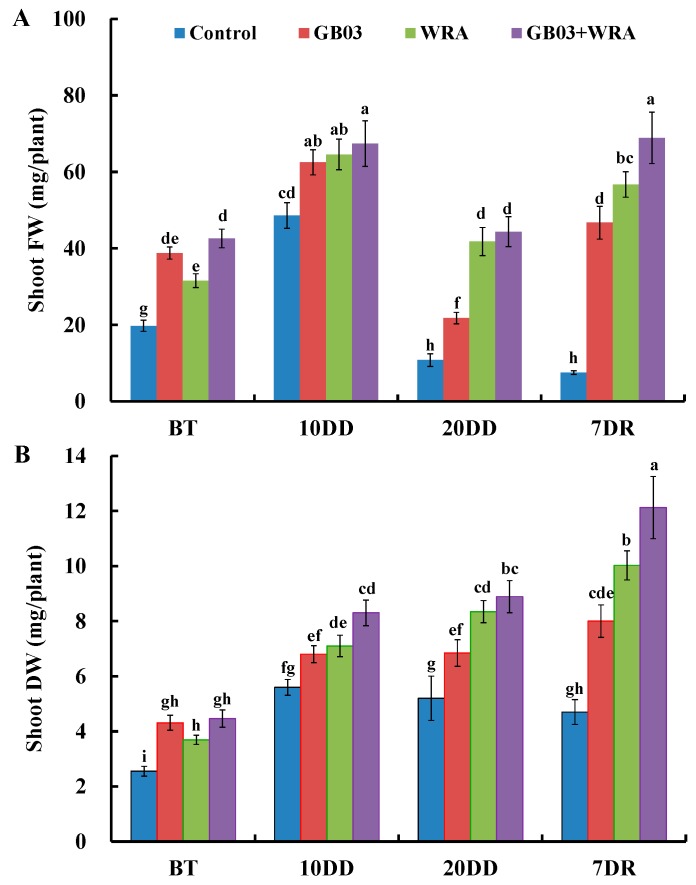
Effects of GB03, water retaining agent (WRA), or the combination of GB03+WRA on shoot fresh weight (FW) (**A**) and dry weight (DW) (**B**) of ryegrass. BT, before treatment (20-day-old seedling); 10DD, 10-day natural drought; 20DD, 20-day natural drought; 7DR, 7 days after rewatering. Values are means and bars indicate SEs (*n* = 12). Columns with different letters indicate significant differences among treatments at *p* < 0.05 (ANOVA and Duncan’s post-hoc multiple comparison test).

After growing for 20 days (BT), GB03 and WRA significantly enhanced shoot fresh weight by 96.3% and 59.7% compared to control, respectively; moreover, the shoot fresh weight of GB03+WRA treatment was 116% and 35.0% significantly higher than that of control and WRA treatment, respectively (Figure 3A). After 10-days of drought treatment, three treatments (GB03, WRA and GB03+WRA) had significantly 28.5%, 32.8%, and 38.6% higher fresh weight than control, respectively. Drought stress triggered significant decreases in shoot fresh weight when seedlings were withheld water for 20 days: all treatments (control, GB03, WRA and GB03+WRA, respectively) decreased by 77.8%, 65.1%, 35.3% and 34.2%. However, in this phase, the fresh weights of GB03 and WRA treatments were significantly 1.02-fold and 2.87-fold higher than that of control, respectively; GB03+WRA treatment was 1.04-fold and 3.11-fold significantly higher in shoot fresh weight than GB03 treatment and control, respectively. Seven days after rewatering, shoot fresh weights for all treatments (except for control) increased significantly than that in 20-day drought. In this phase, three treatments (GB03, WRA and GB03+WRA) were significantly 5.20-fold, 6.52-fold and 8.14-fold higher in fresh weight than control, respectively; the shoot fresh weight in GB03+WRA and WRA treatments were 47.4% and 21.3% significantly higher than that in GB03 treatment, respectively, and GB03+WRA increased shoot fresh weight significantly compared to control and other two treatments (Figure 3A).

Three treatments (GB03, WRA and GB03+WRA) significantly increased shoot dry weight by 68.7%, 44.5% and 74.7% compared to control, respectively, after growing for 20 days (Figure 3B). After 10-day drought, WRA and GB03+WRA treatments increased shoot dry weight significantly by 26.8% and 48.2%, respectively, compared to control. After 10-day drought, three treatments (GB03, WRA and GB03+WRA) were significantly 31.5%, 60.5% and 70.9% higher in dry weight than control, respectively. Seven days after rewatering, WRA and GB03+WRA treatments increased shoot dry weight significantly by 20.1% and 36.4%, respectively, compared to those in 20-day drought treatment. In this phase, three treatments (GB03, WRA and GB03+WRA) were significantly 0.72-fold, 1.13-fold and 1.57-fold higher in shoot dry weight, respectively, compared to control, and GB03+WRA increased shoot dry weight significantly compared to control and other two treatments (Figure 3B).

### 2.2. GB03 and WRA Maintained the Relative Water Content (RWC) under Drought Condition

**Figure 4:**
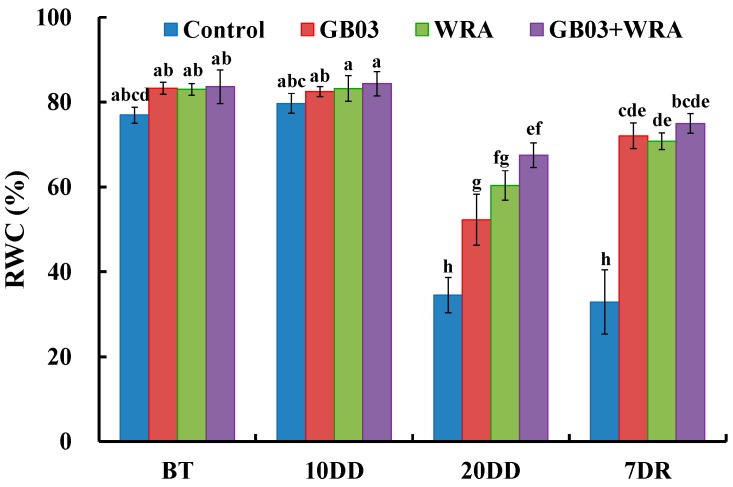
Effects of GB03, water retaining agent (WRA), or the combination of GB03+WRA on leaf relative water content (RWC) of ryegrass. BT, before treatment (20-day-old seedling); 10DD, 10-day natural drought; 20DD, 20-day natural drought; 7DR, 7 days after rewatering. Values are means and bars indicate SEs (*n* = 12). Columns with different letters indicate significant differences among treatments at *p* < 0.05 (ANOVA and Duncan’s post-hoc multiple comparison test).

To probe the plant water status, RWC was assayed in ryegrass leaves. As shown in plant biomass (shoot fresh weight and dry weight), 10-day drought treatment was tolerable for ryegrass with all treatments no significant difference in RWC. After stopping watering for 20 days, all treatments decreased RWC significantly by 56.7%, 36.6%, 27.4% and 20.0%, respectively, compared to those in 10-day drought; the three treatments (GB03, WRA and GB03+WRA) were 51.4%, 74.8% and 95.5% significantly higher in RWC, respectively, compared to control; GB03+WRA treatment was also 29.1% significantly higher than GB03 treatment, indicating that GB03 and WRA together could effectively maintain the RWC in ryegrass. Seven days after rewatering, three treatments (GB03, WRA and GB03+WRA) were significantly 1.19-, 1.15- and 1.27-folds higher than control in RWC, respectively (Figure 4).

### 2.3. GB03 and WRA Maintained Chlorophyll Content

**Figure 5:**
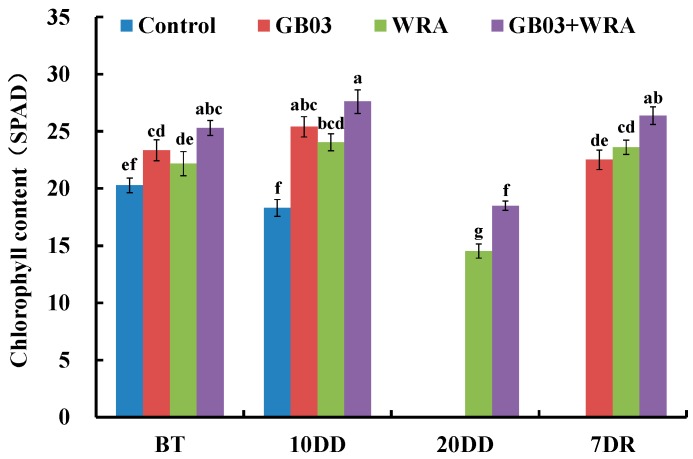
Effects of GB03, water retaining agent (WRA), or the combination of GB03+WRA on leaf chlorophyll content of ryegrass. BT, before treatment (20-day-old seedling); 10DD, 10-day natural drought; 20DD, 20-day natural drought; 7DR, 7 days after rewatering. Values are means and bars indicate SEs (*n* = 12). Columns with different letters indicate significant differences among treatments at *p* < 0.05 (ANOVA and Duncan’s post-hoc multiple comparison test).

After growing for 20 days (BT), chlorophyll content for GB03 and GB03+WRA treatments was 15.1% and 24.8% significantly higher than that in control, respectively; chlorophyll content in GB03+WRA was significantly 14.1% higher than that in WRA. After 10-day drought treatment, chlorophyll content was 38.8%, 31.4% and 50.8% significantly higher in GB03, WRA and GB03+WRA treatments than in control, respectively (Figure 5). After 20-day drought treatment, the chlorophyll content in WRA was 21.4% significantly lower than that in GB03+WRA, however, seedlings with GB03 treatment and control became too wilt and chlorophyll content was unmeasurable. Seven days after rewatering, the chlorophyll content in all three treatments returned to the BT level, while that in control was still beyond measurement. The chlorophyll content in GB03+WRA treatment was 17.1% and 11.7% significantly higher than those in GB03 and WRA treatments, respectively. Therefore, WRA together with GB03 effectively maintained ryegrass chlorophyll content, especially when seedlings were under severe drought stress (20-day drought) (Figure 5).

### 2.4. GB03 and WRA Reduced Relative Electric Conductivity (REC) and MDA Content under Drought Stress

**Figure 6:**
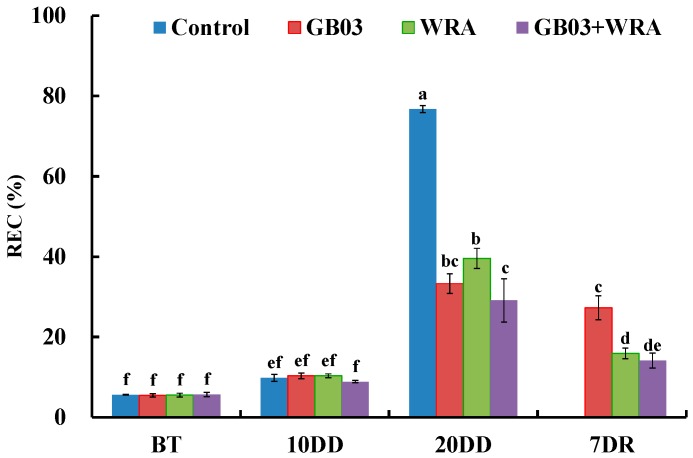
Effects of GB03, water retaining agent (WRA), or the combination of GB03+WRA on relative electric conductivity (REC) of ryegrass. BT, before treatment (20-day-old seedling); 10DD, 10-day natural drought; 20DD, 20-day natural drought; 7DR, 7 days after rewatering Values are means and bars indicate SEs (*n* = 12). Columns with different letters indicate significant differences among treatments at *p* < 0.05 (ANOVA and Duncan’s post-hoc multiple comparison test).

After stopping watering for 20 days, a significant increase in REC was observed compared to 10 days of drought in control and three treatments; REC in control increased drastically by 6.80-fold. However, GB03 and WRA helped seedlings to maintain relatively lower REC and reduced REC significantly by 56.6% and 48.4% compared to control, respectively; moreover, REC in GB03+WRA treatment was 62.1% significantly lower than that in control (Figure 6). These results suggested that GB03 and WRA can ensured the relative low level of REC in ryegrass under severe drought conditions. Seven days after rewatering, REC in WRA and GB03+WRA significantly decreased by 59.8% and 51.5% compared to 20-day drought, respectively; and REC in GB03+WRA was significantly 48.2% lower than GB03. While REC in control plants was beyond measurement (Figure 6).

**Figure 7:**
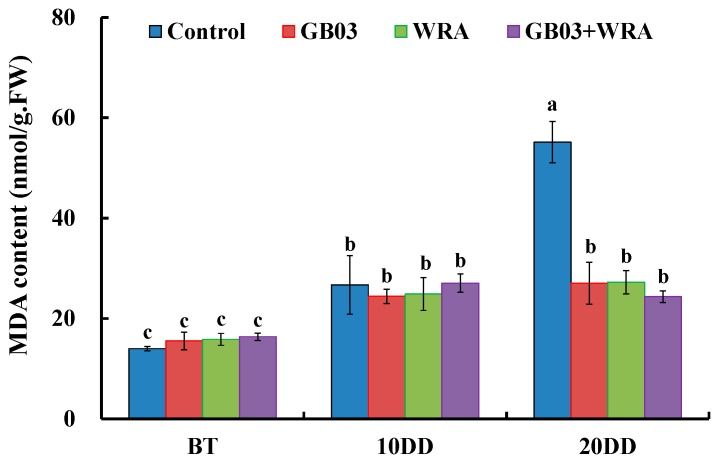
Effects of GB03, water retaining agent (WRA), or the combination of GB03+WRA on leaf malondialdehyde (MDA) content of ryegrass. BT, before treatment (20-day-old seedling); 10DD, 10-day natural drought; 20DD, 20-day natural drought; 7DR, 7 days after rewatering. Values are means and bars indicate SEs (*n* = 12). Columns with different letters indicate significant differences among treatments at *p* < 0.05 (ANOVA and Duncan’s post-hoc multiple comparison test).

After 10-day drought, MDA contents were increased significantly by 90.9%, 57.5%, 57.0% and 65.3% compared with their corresponding BT levels. After 20-day drought, MDA content only in control plants was increased significantly by 1.07-fold compared to 10-day drought, whereas those in the three treatments were still maintained the previous phase level; GB03+WRA treatments was 55.9% significantly lower than control in MDA content (Figure 7).

## 3. Discussion

### 3.1. Synergistic Effects of GB03 and WRA on Plant Growth under Drought Condition

Considerable progress has been made in fathoming mechanisms underlying *Bacillus*-mediated plant growth promotion and crop yield increase; these mechanisms include increased nutrient availability, synthesizing plant hormones and the production of volatile organic compounds [15,16,17,18,19,20]. Plant growth promotion meditated by *Bacillus amyloliquefaciens* has been reported in many species including *Arabidopsis* [15,20,42], maize (*Zea mays* L.) [43], tomato (*Lycopersicon esculentum*) [44], wheat (*Triticum aestivum*) [22], white clover (*Trifolium repens* L. cultivar Huia) [23] and *Puccinellia tenuiflora* [24]. Gagné-Bourque et al. also proved that *B. subtilis* enhanced *Brachypodium distachyon* growth under drought stress [45]. 

Consistent with the recent study of Galeş et al. [34], the soil moisture at the end of the drought treatment showed that the WRA’s property in retaining the water and releasing it afterward (Table 1). Because of this property, treatments amended with WRA in this study positively affected plant growth and the more severe the drought was, the better they performed in comparison to controls (Figure 1 and Figure 2). Similar results for tomato and cucumber were reported by El-Hady and Wanas [46]. The property of WRA, however, is hard to evaluate due to its depending largely on temperature, humidity, the particle size of hydrogel and the properties of the soil [47]. Interestingly, here we found that the combined effect of GB03 and WRA on promoting plant growth was greater than that of either of them.

### 3.2. GB03 and WRA Maintained Relatively Higher RWC Level in Ryegrass Leaves

As one of the best criteria for measuring the water status in plants, RWC indicates the water metabolic activity in tissues as drought-resistant species usually have higher RWC in their leaves [48]. Hence, RWC could also be used as an ideal parameter to probe the PGPR-mediated plant drought tolerance. Indeed, many researchers have reported that under drought stress, plants with PGPR inoculation maintained higher RWC as compared to those without, suggesting that PGPR strains could effectively prolong the plant survival under drought conditions [49,50,51]. In this work, GB03 effectively enhanced the RWC by 51.4% over control under severe drought treatment (20-day drought) (Figure 4), which was consistent with previous research in sorghum [52]. Dodd et al. claimed that the increase in RWC might be a consequence of changes of the sensitivity in stomatal closure [53]. Despite the progress in the recent decade, the mechanisms behind increased RWC with PGPR treatment remain to be elucidated. After stopping watering for 20 days, WRA also elevated the level of RWC in ryegrass by 74.8% over control. This was understandable because WRA retained soil water available for plant. GB03 together with WRA maintained relatively higher RWC level than GB03 or WRA in ryegrass leaves although not significantly.

### 3.3. Synergistic Effects of GB03 and WRA in Ryegrass Leaf Chlorophyll Content under Severe Drought Condition

Leaf chlorophyll content is also an important physiological parameter positively affecting plant photosynthesis rate [7]. As one of the symptoms of photo-oxidation, drought-triggered decrease in chlorophyll content has been observed in maize [54], sorghum [52] and white clover [23]. Zhang et al. found that GB03 increased photosynthetic capacity in *Arabidopsis* by raising photosystem II photosynthetic activity and chlorophyll content [20]. In ryegrass, the increase and maintenance in chlorophyll content by GB03 inoculation were observed under normal condition (BT) and moderate drought condition (10-day drought) (Figure 5). It was observed that WRA enhanced chlorophyll content in maize and soybean crop [33]. With the effect of WRA, the chlorophyll content in this study maintained relatively high level even under severe drought stress (20-day drought). GB03 alone failed to function under severe drought; however, the combination of t GB03 and WRA showed significantly better effect than WRA alone (Figure 5).

### 3.4. GB03 and WRA Alleviated Cell Membrane Damage under Drought Conditions

Drought incurs oxidative stress in plants by increasing ROS that is able to effectively degrade membrane lipids and to exacerbate lipid peroxidation [55,56]. The degradation of cell membrane results in the increase of REC. Besides, with the increase of ROS, the content of MDA follows rapidly. Thus the content of MDA has also been considered as a suitable index of oxidative damage [50,57]. Vardharajula et al. reported *Bacillus* spp. HYD-B17 decreased REC in maize by 26.3% under drought conditions [50]. Similar results were reported by Sandhya et al. [49] and Naveed et al. [51]. In the current study, GB03 and WRA reduced REC significantly in ryegrass under severe drought stress (20-day drought) and prolonged the seedlings’ survivability (Figure 6). As for using MDA as a marker to probe cell damage, Han et al. found GB03 significantly decreased the MDA content in white clover under stress [23]. Here we found similar result in ryegrass under severe drought stress (20-day drought); in addition, we also found that WRA together with GB03 decreased MDA content to the same statistical level as GB03 inoculation and WRA treatments (Figure 7).

## 4. Materials and Methods

### 4.1. Bacterial Suspension Culture

*Bacillus amyloliquefaciens* strain GB03 (presented by Professor Paul W. Paré at Texas Tech University, Lubbock, TX, USA) was streaked onto Luria broth (LB) agar plates and incubated at 28 °C in darkness for 24 h. Bacterial cells were then transferred from LB agar plates to liquid LB medium and cultured under 28 °C and 250 rpm to yield 10^9^ colony forming units (CFU)/mL, as measured by optical density and a series of dilutions [24,58].

### 4.2. Plant Growth and Treatments

Perennial ryegrass (*Lolium perenne* L. cv. Esquire) seeds (Beijing Top Green Seed Co., Ltd., Beijing, China) were surface sterilized with 2% NaClO (sodium hypochlorite) for 1 min followed by 70% ethanol for 10 min, and rinsed by sterilized water five times. Seeds were then grown in pre-sterilized plastic pots (diameter 20 cm, depth 15 cm, 1.5 g seeds/pot with 12 replicates) filled with 1800 g of heat-sterilized (95 °C, 48 h) vermiculite, sand and field top soil mixture with their volume ratio of 1:1:1. Each pot was inoculated with 10 mL GB03 suspension culture or 10 mL liquid LB medium. Then, half of each group of above two treatments was applied WRA (5 g/pot, the compound is super absorbent hydrogels provided by Zhuhai Nongshen Biotechnology Co., Ltd., Zhuhai, China). Thus, four treatments were set: control, GB03, WRA and GB03+WRA. Each treatment contained 12 replications (12 pots) and all 48 pots were daily rearranged to avoid any border effect or light heterogeneity. Each pot was irrigated with tap water (250 mL) every 3 days until drought treatment started. Plants were grown in greenhouse under 28 °C/23 °C (day/night), the photoperiod was 16/8 h (light/dark) and the relative humidity was about 70%.

Twenty-day-old plants were subjected to drought stress by stopping watering for 20 days, and then regular watering was continued. Seedlings were harvested on the 0th (BT), 10th and 20th day after stopping watering and 7th day after rewatering for plant biomass and physiological measurements. Soil samples were taken to measure soil moisture on the 20th day after stopping watering (Table 1).

### 4.3. Plant Survival Rate, Biomass and Physiological Measurements

The numbers of survived plants and dead plants in each pot were counted after 20 days natural drought and then and survival rate (%) was calculated (*n* = 12).

Two plants from each of the 12 pots (12 replications) at each stage were sampled to measure biomass and physiological indexes. Shoot fresh weight was measured at once after harvest. Turgid weights of leaves were measured after they were soaked in distilled water in test tubes at 4 °C overnight in the dark. Finally, shoots and leaves were dried in an oven under 80 °C for 48 h and weighed again to get shoot and leaf dry weight. Leaf relative water content (RWC) was estimated according to the method described by Barrs and Weatherley [59] and then calculated using the following equation, where FW represents the leaf fresh weight, TW the leaf turgid weight and DW the leaf dry weight.

$$\mathrm{RWC}\;(\%)\;=\;\frac{FW-DW}{TW-DW}\times 100$$

Total chlorophyll content was measured by using chlorophyll meter (SPAD 502, Konica Minolta Sensing, Inc., Osaka, Japan).

Leaf relative electric conductivity (REC) was measured to estimate leaf cell membrane damage using an electric conductivity meter (EC215, Hanna Corporation, Italy) as described by Peever and Higgins [60] and Niu et al. [61] with slight modifications. REC (%) was calculated using the following equation, where S1 and S2 refer to conductivity of ryegrass live leaves and boiled leaves, respectively.

$$\mathrm{REC}\;(\%)\;=\;\frac{S1}{S2}\times 100$$

To probe leaf oxidative damage, the biomarker malondialdehyde (MDA) was extracted and determined spectrophotometrically using a thiobarbituric acid (TBA) protocol [23]. Reagent kit was supplied by Suzhou Comin Biotechnology Co., Ltd. (Suzhou, China) Absorbance was determined at 532 and 600 nm using a UV spectrophotometer (UV-2102C, Unico Instrument Co., Ltd., Shanghai, China).

### 4.4. Data Analysis

Results of the growth and physiological parameters were presented as means with standard errors (*n* = 12). All the data were subjected to one-way analysis of variance (ANOVA) and Duncan’s post-hoc multiple comparison tests were used to detect significant differences among means at a significance level of *p* < 0.05 using SPSS 17.0 (SPSS Inc., Chicago, IL, USA).

## 5. Conclusions

This study demonstrated that either GB03 or WRA promotes ryegrass survival and growth under drought conditions by directly or indirectly maintaining survival rate, biomass, relative water content, leaf chlorophyll content and cell membrane integrity. Furthermore, the synergistic effect of GB03 and WRA on drought tolerance promotion of ryegrass were greater than that of either of them by improving survival rate, biomass and chlorophyll content. This work could be helpful to develop a fresh and excellent approach for turf to cope with the challenge of global fresh water insufficiency.

## Figures and Tables

**Table 1:** Soil water content after 20 days of natural drought. Values are means with SEs (*n* = 12). Different letters indicate significant differences among treatments at *p* < 0.05 (ANOVA and Duncan’s multiple range test).

Treatments	Control	GB03	WRA	GB03+WRA
Soil water content (mg/g·DW)	37.7 ± 3.9 b	33.3 ± 04.7 b	61.8 ± 2.0 a	62.4 ± 5.9 a

## Abbreviations

BT	Before drought treatment
CFU	Colony Forming Units
LB	Luria Broth
MDA	Malondialdehyde
PGPR	Plant Growth Promoting Rhizobacteria
REC	Relative electric conductivity
RWC	Relative Water Content
SEs	Standard errors
VOCs	Volatile Organic Compounds
WRA	Water Retaining Agent
